# Training for happiness: the impacts of different positive exercises on hedonism and eudaemonia

**DOI:** 10.1186/s40064-016-2407-y

**Published:** 2016-06-16

**Authors:** Miguel Pereira Lopes, Patricia Jardim da Palma, Bruno Cardoso Garcia, Catarina Gomes

**Affiliations:** School of Social and Political Sciences, Lisbon University, Rua Almerindo Lessa, 1300-663 Lisbon, Portugal

**Keywords:** Hedonic enjoyment, Happiness, Eudaemonia, Experimental, Positive exercise

## Abstract

Theoretical conceptions on happiness have generally considered two broad perspectives: hedonic enjoyment and eudaemonia. However, most research on how to improve people’s happiness has focused primarily on the enhancement of hedonic happiness. In this longitudinal experimental study we test the differential impact of two positive exercises—Best Possible Selves and the Lottery Question—on hedonic and eudaemonic happiness. The hypothesis that the practice of the Best Possible Selves exercise would increase hedonic happiness was confirmed. This effect was immediate and maintained a week after the exercise. Furthermore, this exercise also increased eudaemonic happiness. However, its effect decreased after a week. Contrary to what was expected the Lottery Question exercise decreased both eudaemonic happiness and hedonic happiness over time. We discuss implications of this study for the literature on positive psychological and behavioral interventions to increase happiness.

## Background

Hedonic and eudaemonic happiness have been described with reference to the two Greek mythological entities of Dionysius and Apollo, the first mirroring an orientation to pleasure and the second as representative of an orientation to meaning of life (Linley and Leontiev 2009). Hedonism regards happiness as consisting of subjective happiness and concerns the experience of pleasure versus displeasure broadly construed to include all judgements about the good and bad elements of life (Ryan and Deci 2001). A key example of this approach is reflected in the name of a fundamental book on the filed called “Well-being: Foundations of hedonic psychology” (Kahneman et al. 1999). On the other side, eudaemonism regards happiness as the actualization of human potentials, of fulfilling or realizing one’s daemon or true nature—that is, of fulfilling one’s virtuous potentials and living as one was inherently intended to live (Deci and Ryan 2008; Ryff 1989).

These two perspectives mirror different views of human nature. Whereas the hedonic approach considers the human organism to be relatively empty and thus malleable (Tooby and Cosmides 1992), the eudaemonic approach ascribes content to human nature and works to uncover that content and to understand the conditions that facilitate versus diminish it (Deci and Ryan 2008).

One of the most controversial aspects in this stream of research relates to the fact that there is scarce scientific support for the idea that people’s level of happiness can change for the better (Lyubomirsky et al. 2005). These authors advance that very few interventions on happiness studies have been carried out to understand the “possibility of becoming happier”. In addition, the conception of happiness that underlies the existing studies is not always explicit.

 Another controversy is about the fact that the hedonic view still predominates in most applied research concerning happiness (Deci and Ryan 2008). Interventions studies on happiness are relatively recent and have not placed any emphasis on the distinction between hedonic and eudaemonic happiness. As pointed out by Lyubomirsky et al. (2005), some research indicating positive effects of prompting people to practice positive psychological “virtues” such as gratitude, hope and forgiveness suggest that cognitive activity offers many excellent possibilities for happiness interventions. Yet, there is sparse research on the application of positive interventions to explicitly address differences between hedonism and eudaemonism.

As such, having in mind the two conceptions of well-being (hedonic enjoyment and eudaemonia) and the ways in which these differ and overlap, the goal of this study is to test the impact of different positive exercises on different types of happiness. With that purpose in mind, we had participants practicing an exercise either in line with the hedonic view of well-being or in line with the eudaemonic view specially created for this study. The effects of these two exercises were studied while controlling for the levels of pre and post measures of hedonic and eudaemonic happiness. Thus, the study focused on the effects of specific exercises on different kinds of happiness over time.

The study has several contributions to the literature. First, it proposes and tests the impact of a new and distinct positive intervention exercise that can be used in practice. The test of positive behavior interventions has become an accepted field of research but there is still much to learn about these matters. Second, the present study contributes to the advancement of this field of research also by testing the differential impacts of different kinds of exercises. Third, by understanding how to promote different happiness outcomes (i.e., hedonic and eudaemonic) the present study contributes to a better knowledge of different kinds of happiness work and how they can be achieved.

The remaining of this article is structured as follows. First, an overview of the two conceptions of happiness is briefly discussed in relation to positive exercises. We then present the methodological approach underlying this study. After, we describe the results in detail with a special focus on hypothesis testing. Finally, we provide a critical analysis of the results along with the contributions and main future directions of research.

## Theory and hypothesis

As introduced earlier, two broad conceptualizations have commonly been used to address the topic of happiness: hedonism, which focuses on happiness and defines well-being in terms of pleasure attainment and pain avoidance; and eudaemonism, which focuses on meaning and self-actualization and defines well-being in terms of the degree to which a person is fully functioning (Ryan and Deci 2001).

This conceptual distinction has raised controversy concerning its validity and usefulness (Kashdan et al. 2008), mostly because hedonism and eudaemonia overlap, despite their differences. Yet, over the last years, several researchers have advanced empirical findings, establishing both the validity and usefulness of the distinction between hedonic happiness and eudaemonia, while still recognizing that both are strongly associated (Waterman et al. 2008).

Research reflecting the notion that the distinction between eudaemonia and hedonic happiness is desirable comes from McGregor and Little (1998). These authors analyzed a diverse set of mental health indicators and also found two fundamental factors, one reflecting hedonic happiness and the other, meaningfulness or eudaemonic happiness. These researchers showed that, when pursuing personal goals, doing well and feeling happy may be disconnected from finding meaning and acting with integrity. This research alludes to another key concept in understanding well-being: meaning in life. According to Steger (2012), meaning in life is characterized as being comprised of people’s comprehension of the world around them and their investment in a self-concordant purpose. Frankl (1963) argued that humans are characterized by a ‘will to meaning’, highlighting the search for meaning as human’s primary motivation in life. Steger et al. (2006), consider meaning to be of critical importance for eudaemonic theories of well-being, since it is either a fundamental component or a result of maximizing one’s potentials.

Keyes and Annas (2009) have mentioned several studies that statistically support that hedonic and eudaemonic measures of well-being are not redundant conceptually or empirically. Despite the significant overlap between these two views, we conclude that those aspects highlighting divergence rather than just convergence in the hedonic and eudaemonic measures of happiness, may result in relevant analyses.

### Positive behavior intervention studies

The debate of whether is it possible to intervene in happiness levels is tainted by considerable scientific pessimism (Lyubomirsky et al. 2005), which is founded in three different ideas: first, the idea of a genetically determined set point for happiness; second, the fact that personality traits (being cognitive, affective and behavioral complexes) are by definition consistent across situations and time, accounting for part of the stability of the set point; third, the concept of “hedonic treadmill”, which suggests that any gains in happiness are only temporary, because humans adapt quickly to change.

Despite this pessimism, some authors have advanced good reasons to pursue an answer to this question. For instance, Sin and Lyubomirsky (2009) have conducted a meta-analysis of 51 positive psychology interventions (PPIs) and have concluded that these interventions do enhance happiness significantly. This research argues that PPI strategies as diverse as writing gratitude letters, practicing optimistic thinking, replaying positive experiences, and socializing have been shown to increase well-being and happiness in nonclinical samples (e.g., Fordyce 1977; Lyubomirsky et al. 2011; Ruini et al. 2006).

Although the research discussed in the present study clearly demonstrate that intervention studies are of critical importance to understand and influence happiness, it is less clear that these intervention studies have covered both the hedonic and the eudaemonic perspectives. In fact, in the architecture of the sustainable happiness model advanced by Lyubomirsky et al. (2005) to support intervention studies, the authors considered a definition of happiness in terms of frequent positive affect, high life satisfaction and infrequent negative affect (a definition related to subjective well-being) which hints at the notion that these interventions focus primarily in the hedonic perspective of happiness.

Our aim is to test the application of different positive intervention exercises (for both hedonic and eudaemonic happiness), and to test their differential impacts on both types of happiness. To positively influence hedonic happiness we have considered the practice of visualization of the best possible self (Best Possible Selves). Best Possible Selves has been defined as an idiographic representation of goals (Markus and Nurius 1986), encompassing all of the futures that people can imagine for themselves. It is usually regarded as a source of benefits likely to contribute to increase and sustain positive affect which is one of the components of SWB (i.e., the hedonic perspective of happiness).

To positively influence eudaemonic happiness we developed a new positive psychological exercise named Lottery Question, based on previous scientific research (Highouse et al. 2010; Vecchio 1980). That research stream strives to better understand what is behind the human motivation to work and is concerned with the meaning of work and life and the work ethic issue (Weber 1930). Although in most of these studies participants are simply asked to think about what they would do if they got enough money to stop working and still live financially comfortable, their methodology has become known as the “lottery question” because in some of these studies the data relies on a question that asked participants to think about what they would do if they actually win the lottery (Highouse et al. 2010; Kaplan 1987).

Waterman (1993) asserted that happiness is usually defined in a hedonic way. However, the eudaemonic conception of happiness calls people to live in accordance with their own daemon, or true self. As such, our assumption is that the Lottery Question that was developed in this study and described in the following section invokes a reflection on the ideal circumstances in which a person can be her true self, or can devote himself to those activities that are more profoundly fulfilling. Thus, anchored by two practices assumed to tap into distinct types of happiness (the Best Possible Selves to develop hedonic happiness and the Lottery Question to develop eudaemonic happiness), we have attempted to test the impact of different positive exercises on hedonic and eudaemonic well-being.

Having in mind the previous literature and also the longitudinal approach of this study, the following hypotheses were developed:

#### **H1**

The Best Possible Self exercise leads to an increase in hedonic happiness, but not in eudaemonic happiness.

#### **H2**

The Lottery Question exercise leads to an increase in eudaemonic happiness but not in hedonic happiness.

### Momentary and follow-up impacts of positive interventions

Another point of analysis in the present study is the effect of hedonic happiness and eudaemonia over time. As mentioned above, there is some research on the longitudinal effects of positive exercises. For example, in a study carried out by Sheldon and Lyubomirsky (2006) participants were asked to keep performing one of a few affect boosting exercises over a period of several weeks, and then measure how conscientiously they did it. In that research the Best Possible Selves exercise did show a significant increase in immediate positive affect. Yet, the participants who performed this exercise only managed to maintain their positive affect when exercise performance was taken into account and, no lasting effects of the exercise alone were found on follow up. As in most research undertaken concerning the effects of positive exercises over time, this research considered only aspects related to hedonic happiness. Our goal is to include also aspects specifically related to eudaemonia.

Our rationale was that eudaemonia is more than a psychological state of mind. It refers more closely to how a person is functioning and is closely related with meaning in life which in turn evokes a longer-term compromise. This led us to consider the possibility that exercises with an effect on eudaemonia are more likely to subsist over time.

Given this, the following hypotheses can be outlined:

#### **H3**

The Best Possible Self exercise leads to an immediate increase in hedonic happiness but reduces its effect a week after its practice.

#### **H4**

The Lottery Question exercise leads to an immediate increase in eudaemonic happiness and sustains its effect a week after its practice.

## Methods

### Design and participants

A simple experiment was carried out longitudinally in order to test the longitudinal effect of two different exercises in two groups. A total of 137 undergraduate students enrolled in a same social sciences class, from a major Portuguese University, were recruited to participate in this study. However, only 62 individuals accepted to participate throughout all the study. These participants corresponded to 45.3 % of all the recruited students. In average the participants were 28.4 years old (SD = 9.2) and 57.1 % of them were females. Regarding the two groups, one comprised 38 participants and the other 24 participants.

### Procedure and exercises

All students were asked to participate voluntarily in the study and, were informed that the study regarded happiness. After we had their consent, guaranteed the anonymity and confidentiality of their data and explained to them the possibility of withdrawal, we proceeded with the collection of data.

Participants were randomly assigned to do one of two exercises: the Best Possible Selves and the Lottery Question.

#### The Best Possible Selves

The Best Possible Selves exercise, as described in Sheldon and Lyubomirsky (2006), was introduced in writing as follows: *“Think about your best possible self” means that you imagine yourself in the future, after everything has gone as well as it possibly could. You have worked hard and succeeded at accomplishing all of your life goals. Think of this as the realization of your life dreams, and of your own best potentials. In all of these cases you are identifying the best possible way that things might turn out in your life, in order to help guide your decisions now.*

Participants who were randomly assigned the Best Possible Selves exercise (n = 38), were directed to write freely about a maximum of 20 min about their “ideal life in the future” in a blank space of several lines provided. They were prompted to outline their “ideal future life” in as much detail as they could.

#### The Lottery Question

The Lottery Question exercise was created based on the work of Highouse et al. (2010) and Vecchio (1980) for this study and was introduced in writing as follows: “Imagine that you had the possibility to live without financial worrisome. Imagine, for instance, that you have won many millions in the lottery or that you have inherited enough money to do whatever you want in life without working. Please, think deeply about this situation and after a genuine reflection describe in the following lines what would you do in your life. Think in a typical day as an example and write as detailed as possible how you would occupy your day. Perhaps in an initial stage you would like to make your lifetime dreams come true, including buying the car or the house of your dreams or making the trip of your life. However, we are interested in knowing how you would occupy your time after that initial stage, when all your material dreams were completed.”

Participants who were randomly assigned the Lottery Question exercise (n = 24), were directed to write freely about a maximum of 20 min about their “meaning of life and work” in a blank space of several lines provided. They were prompted to outline their “meaning of life and work” in as much detail as they could.

In line with Sheldon and Lyubomirsky (2006) both exercises were verbally introduced by the experimenter and, were preceded with the following statement: “In this study we are studying happiness, and what sustains it. We will assess your happiness three times during this semester, to see how it fluctuates. We will also ask you to do an exercise during this time that might affect your happiness. Research suggests that this exercise has already been shown to have significant positive effects on peoples’ lives, and we intend to further understand its potential.” Also, in line with the authors, in order to encourage the participants’ commitment to perform the exercises, they were informed of the purpose of the exercises (i.e., influencing happiness). A possible bias of the results was prevented because all participants in the two conditions received the same verbal message and indications regarding the purpose of the exercises.

The whole study comprised 6 moments each in 6 different days. Moments 1–5 occurred in five consecutive days and moment 6 occurred 1 week after moment 5. Participants were surveyed at the same time in 3 moments: moment 1 (T1)—pre intervention—, moment 5 (T2)—post-intervention—and, moment 6 (T3)—follow up. In these moments (T1, T2, and T3) all participants responded to the same surveys, which included both measures of hedonic and eudaemonic happiness. In line with Sheldon and Lyubomirsky (2006) during the period between T1 and T2 (moments 2, 3 and 4), the participants filled in for a maximum of 20 min the two different positive psychological exercises, the Best Possible Selves and the Lottery Question.

In order to assure response anonymity, each respondent was asked to fill in the same alphanumerical code in all the questionnaires and materials throughout the whole 2 weeks during which the experiment was run, therefore making it possible to pair individual responses across all moments. A week after moment 6 (T3), participants were debriefed about all the goals of the study, were thanked for their collaboration and were encouraged to keep exercising both the Best Possible Selves and the Lottery Question exercises.

### Measures

This study gathered two self-report instruments following the literature on hedonic and eudaemonic happiness. We relied on a measure of positive affect as a proxy to hedonic happiness, as it refers to the positive emotional experience in the moment. As for eudaemonic happiness, we relied on a measure of purpose in life, one of its core characteristics. The psychometric characteristics were tested for all the self-report measures. Regarding these instruments these were translated into Portuguese by two experienced researchers, fluent in English. The Portuguese version of it was later translated back to English, by another native English speaker into English language to ensure the accuracy of the translation, warranting, therefore the items’ linguistic particularities.

#### Positive affect

Positive affect was measured using PANAS (Watson et al. 1988). This measure consists of two 10-item mood scales and was developed to provide brief measures of Positive Affect and Negative Affect. Given the scope of the present paper we focused on the Positive Affect subscale. Given this, respondents were asked to rate the extent to which they had experienced each particular emotion (e.g., “interested”, “excited”) within the last days. All items were scored on a 7-point rating scale ranging from 1 “Never” to 7 “Always”.

Firstly, an initial Exploratory Factor Analysis (EFA) was performed to the 10 original PA items to explore the existence of an “underlying structure” measuring the present subscale at the three different time points. The extraction of factors was based on principal components analysis conducted with varimax (Costello and Osborne 2005). Items selection was achieved through a significant loading cut-off of .50 based on “pragmatic reasoning” (Yong and Pearce 2013). The initial analysis retained only one component, using Kaiser’s criteria (components with eigenvalues above 1 should be retained). After rotation, it revealed a single factor structure with 7 items at all three time points, accounting for 51.9, 54.2 and 70 % of the explained variance respectively. Secondly, a Confirmatory Factor Analysis (CFA) was carried out using the Lavaan package, for the R system, a software for statistical computing (Rosseel 2012), the data revealed a good fit for all three time points: T1 [χ^2^(14) = 18.813, *p* = .172, RMSEA = .074, CFI = .967, TLI = .950 and SRMR = .060], T2 [χ^2^(14) = 34.059, *p* = .002, RMSEA = .152, CFI = .885, TLI = .827 and SRMR = .064] and T3 [χ^2^(14) = 15.730, *p* = .330, RMSEA = .045, CFI = .994, TLI = .991 and SRMR = .032]. This 7 items measure presented a good internal consistency in all time points (α_T1_ = .835; α_T2_ = .849 and α_T3_ = .928).

#### Purpose in life

To measure purpose in life we used the Purpose in Life subscale from Ryff (1989) psychological well-being instrument. To accommodate time and sample restrictions we followed Ryff and Keyes (1995) suggestion to chose only 3 of the original items to measure this construct. This subscale had 3 items (e.g., “I hold beliefs that give life a purpose”). Respondents were asked to rate the extent to which they agreed with each of the items, with reference to a 7-point scale, ranging from 1 ‘Totally Disagree’ to 7 ‘Totally Agree’. Again, throughout the EFA a single factor was found explaining 67.1, 69.4 and 75.2 % of the total variance in all time points, respectively. Also the one-factor model with three items revealed a good fit through CFA analysis, the same throughout all time points [χ^2^(0) = .00, *p* = 0, RMSEA = .000, CFI = 1, TLI = 1 and SRMR = .000]. The Cronbach’s alpha coefficient was (T1) .717, (T2) .756 and (T3) .829.

## Results

To test Hypothesis 1, 2, 3 and 4 a repeated measures ANOVA was conducted. It intended to determine if there were significant differences between the groups regarding the Best Possible Selves and Lottery Question exercises and their impact on each measure of happiness [hedonic happiness (Positive Affect) and eudaemonic happiness (Purpose in Life)] along time. As such exercise type (Best Possible Selves vs Lottery Question) was a between-subjects factor with two levels and, time of measurement [pre-intervention (T1) vs post-intervention (T2) vs follow-up (T3)] was a within-subjects factor with three levels.

In order to proceed with the analysis a Mauchly’s Test of Sphericity was conducted. It indicated that the assumption of sphericity had not been violated along time in both groups regarding their happiness measurements, χ^2^(2) = 5.69, *p* > .05 and χ^2^(2) = 5.53, *p* > .05, and therefore, sphericity was assumed.

The results of the between subjects’ analysis indicated that there were no statistically significant differences between the groups [Best Possible Selves (BPS) vs Lottery Question (LQ)] regarding overall happiness values (M_PILLQ_ = 5.49, DP = 0.15; M_PILBPS_ = 5.24, DP = 0.12; M_PALQ_ = 5.09, DP = 0.12; M_PABPS_ = 4.86, DP = 0.10) [F(2, 59) = 1.28, p > .05; Wilk’s Λ = 0.96, partial η^2^ = .04].

However, the tests of within-subjects’ effects revealed a main effect of time and a significant interaction effect between time and the exercises carried out (Best Possible Selves vs Lottery Question) on the participants hedonic and eudaemonic happiness indicators.

On the one hand the tests within subjects indicated that there were significant differences regarding the measurement of happiness along time [F(4, 57) = 9.6, p < .05; Wilk’s Λ = 0.59, partial η^2^ = .40]. Specifically, post hoc tests using the Bonferroni correction revealed that purpose in life tended to significantly decrease from T1 to T3 and again from T2 to T3 (M_PILT1_ = 5.61, DP = 0.12; M_PILT2_ = 5.56, DP = 0.11; M_PILT2_ = 4.92, DP = 0.12).

**Fig. 1:**
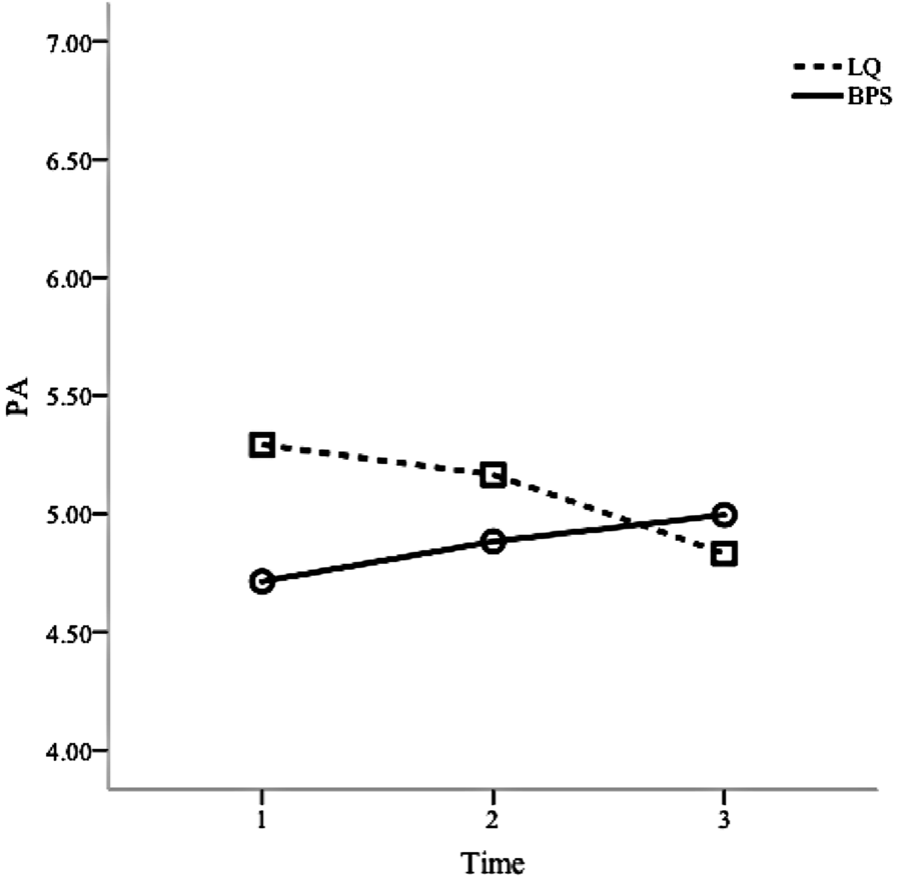
Hedonic [Positive Affect (PA)] levels throughout time regarding the Best Possible Selves (BPS) and Lottery Question (LQ) exercises

**Fig. 2:**
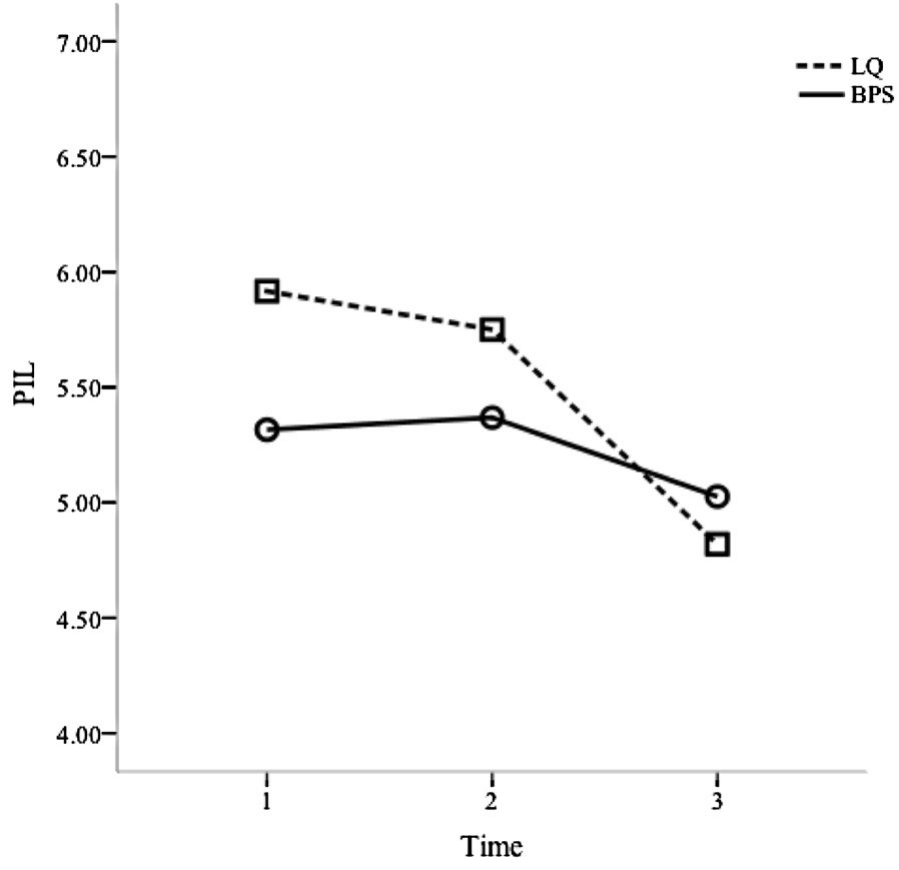
Eudaemonic [Purpose of Life (PIL)] levels throughout time regarding the Best Possible Selves (BPS) and Lottery Question (LQ) exercises

On the other hand, the tests within subjects also showed that there was a significant interaction effect between time and both exercises on happiness measures along time [F(4, 57) = 3.4, p < .05; Wilk’s Λ = 0.80, partial η^2^ = .19]. Follow-up analyses of within-subjects contrasts revealed this interaction to be linearly significant for both types of happiness: eudaemonic happiness (F_PIL_(1) = 8.67, p < .05) and hedonic happiness (F_PA_(1) = 4.03, p < .05). This result indicates that the happiness ratings along time were different for the Best Possible Selves and Lottery Question exercises. Table 1 presents the means and standard deviations for pre-intervention (T1), post-intervention (T2), and follow-up (T3) happiness scores by exercise (Best Possible Selves vs Lottery Question). Figures 1 and 2, respectively, illustrate the hedonic (positive affect) and eudaemonic (purpose of life) levels throughout time regarding the two different exercises.

**Table 1:** Means and standard deviations for pre-intervention (T1), post-intervention (T2), and follow-up (T3) happiness [Positive Affect (PA) and Purpose in Life (PIL)] scores by exercise

Exercise	Mean	SD	N
BPS			
PA (T1)	4.71	0.86	38
PA (T2)	4.88	0.74	38
PA (T3)	5.00	0.63	38
PIL (T1)	5.32	1.11	38
PIL (T2)	5.37	0.86	38
PIL (T3)	5.03	0.89	38
LQ			
PA (T1)	5.29	0.65	24
PA (T2)	5.17	0.61	24
PA (T3)	4.83	0.89	24
PIL (T1)	5.92	0.63	24
PIL (T2)	5.75	0.76	24
PIL (T3)	4.82	1.03	24

Considering the within-subjects’ results presented in the above mentioned Table 1 and Figs. 1 and 2, it can be seen that the Best Possible Selves exercise leads to an increase in hedonic happiness (Positive Affect) but it also leads to an increase in eudaemonic happiness (Purpose In Life) from T1 to T2. As such, these results partially corroborate Hypothesis 1. Also, Best Possible Selves leads to an initial increase in hedonic happiness (Positive Affect) however, it tends to increase its effect after a week (T3). In this sense, these results partially confirm Hypothesis 3, given that it was expected that the Best Possible Selves effect on Positive Affect would be reduced after a week.

Regarding the Lottery Question exercise and, again considering the data on Table 1 and Figs. 1 and 2, this exercise tends to lead to a decrease in eudaemonic happiness (Purpose in Life) and hedonic happiness (Positive Affect) from T1 to T2. Following this, Hypothesis 2 is rejected. Furthermore, contrary to what was expected, the Lottery Question exercise leads to a decrease along time in eudaemonic happiness (Purpose in Life) and sustains it after a week (T3). Given this, Hypothesis 4 is also rejected.

## Discussion

The goal of this study was to test the application of different positive intervention exercises (for both hedonic and eudaemonic happiness), and to test their differential impacts on both types of happiness. Evidence indicates that different positive behavior exercises can have different impacts on different kinds of happiness.

As predicted, in Hypothesis 1, the Best Possible Selves exercise positively impacted on hedonic happiness. This result was in line with previous research (Sheldon and Lyubomirsky 2006), reinforcing the effect of such exercise on hedonic happiness. However, it extended Sheldon and Lyubomirsky (2006) study in two ways.

First, regarding hedonic happiness, the results indicated that this exercise further had an effect on follow up, contrary to what was expected (H3). Considering this, and contrary to what had been supposed by the authors (Sheldon and Lyubomirsky 2006) to happen in college populations, in this case this exercise might have been interpreted as a means of becoming happier, opposed to interpreted as a buffer or source of resilience.

Secondly, regarding eudaemonic happiness, an immediate increase was also observed (from T1 to T2) when practicing the Best Possible Selves exercise, however it decreased a week later (T3). This result, although not expected, may indicate that an overlap between eudaemonic and hedonic happiness can be stimulated by this kind of intervention, as both tend to increase when this exercise is applied. It further, indicates that this exercise does not allow for follow up maintenance regarding eudaemonic happiness. Following this, and in line with Waterman et al. (2008), this result suggests that these two types of happiness might be associated, as they both can be momentarily stimulated by the Best Possible Selves exercise, however, they behave differently along time, indicating distinction between them. Future research should better investigate if this is so and what mechanisms relate to this dual but still differential impact of the Best Possible Selves exercise.

Contrary to what was expected, both Hypothesis 2 and 4 were rejected, regarding the proposed Lottery Question exercise. The new Lottery Question positive exercise developed for this study, based on work by Highouse et al. (2010) and Vecchio (1980) negatively impacted eudaemonic happiness and hedonic happiness along all three time points. These results might be explained by cultural characteristics of the Portuguese sample regarding the exercise. According to Hofstede’s (1991) indulgence dimension, Portuguese individuals tend to be pessimists; they do not put much emphasis on leisure time and control the gratification of their desires. Individuals with this orientation perceive their actions as being restrained by social norms and feel that indulging themselves is somewhat wrong. Following this, the study participants could have interpreted that assuming their true selves was somewhat wrong along the exercise, and also along time. Future research should control for cultural characteristics that might play a part in influencing happiness levels.

### Cultural issues

A core conclusion from this study is that hedonic and eudaemonic happiness can be momentarily enhanced by practicing positive psychological exercises, namely the Best Possible Selves exercise, however, they tend to differ on follow up. The tradition in studying positive exercises has mainly emphasized the enhancement of increases on hedonic happiness (e.g., Lyubomirsky et al. 2005), but that might be the consequence of a bias resulting from a western perspective of happiness, particularly from US scholars. In fact, criticisms concerning a biased western American-led perspective of happiness have long been pointed to the positive psychology movement (Held 2004), and intercultural research in positive behavior exercises and interventions is scarce. However, this study shows that intercultural research in this field might be necessary to better understand how different exercises and interventions are understood and impact the behavior of people in different cultural settings. Research has found that people from different cultures value different aspects of their well-being and happiness (Lau et al. 2005). Research investigating different “happiness profiles” in different countries have been done. For instance, Park et al. (2009) found three different clusters of countries according to measures of pleasure, engagement, meaning, and life satisfaction, showing that different cultures might value different aspects of what happiness is. Nevertheless, this intercultural-sensitive research (Linley and Leontiev 2009) has not yet been explicitly studied concerning positive behavior interventions. As such, if there is a cultural bias, and participants value hedonic happiness more than eudaemonic happiness, respondents might be more direct and immediately influenced by the Best Possible Selves exercise and might even have more difficulty in thinking about their *daemon*, or true self.

### Temporal issues

A final important point raised by this study relates to the temporal issues in positive behavioral interventions and happiness. The sustainability of positive behavior interventions has been a critical issue in the happiness research (Lyubomirsky et al. 2005), with researchers investigating the momentary and follow up impacts of different interventions on several measures of well-being. A consistent conclusion has been that sustaining happiness needs the continued practice of positive exercises (e.g., Cohn and Fredrickson 2010). For instance, the model of the architecture of sustainable happiness proposed by Lyubomirsky et al. (2005) incorporates more or less implicitly that the practice of positive psychological interventions should be continued in time (both with intentional activities or selecting certain circumstances) in order to avoid a return into a person’s initial happiness set point. This is, again, aligned with a western and hedonic perspective of happiness. However, in the present study, we found evidence for the argument that when positive interventions aim to increase hedonic happiness, the positive effect can become sustained without further immediate interventions. Future studies should extend this line of research by testing the impacts of these exercises with further longitudinal time-frames, particularly long-term effects.

### Limitations

Despite these relevant findings, the present study has some limitations. A main limitation is the final sample and subsamples that considered only the participants that have gone throughout the whole study. This is indeed a current problem of longitudinal research and the sample of this study is not different in size from similar studies (e.g., Sheldon and Lyubomirsky 2006). Similar future research following the logic of this study with both equivalent and different samples sizes should be of great value.

Another limitation is the use of self-reported measures, which could lead to common method bias. Although the longitudinal design helps to overcome this bias, given that previous levels of the variables are controlled for to a certain degree, future studies should consider also the use of objective data (e.g., physiological measures), particularly when it comes to happiness.

Also, the non-use of a control group where no intervention would be implemented may be a limitation. As this was an experimental study, a control group could have further contributed for the improvement of the interaction differences regarding the interventions. Future studies should take this into consideration.

The study also has the limitation of including the use of a single sample of undergraduate students, a fact that does not allow us to conclude if there is any cultural bias behind these results. Although again this is a normal shortcoming of similar studies, future research should systematically address the issue of intercultural differences in studying the impacts of positive psychological and behavior interventions.

## Conclusion

The present study contributes to the literature on positive behavior interventions by critically analyzing the bias of positive psychological exercises towards considering happiness as hedonic happiness only. The present study develops this field of research and improves the knowledge of how different types of positive exercises differently impact on different kinds of happiness (hedonic happiness and eudaemonic happiness). Following this, future research should continue to investigate how each of the positive exercises differently influences different happiness outcomes, particularly considering the cultural contexts of where the exercises are being practiced.
